# Rab4b Promotes Cytolethal Distending Toxin from *Glaesserella parasuis*-Induced Cytotoxicity in PK-15 Cells

**DOI:** 10.3390/toxins16090407

**Published:** 2024-09-19

**Authors:** Yiwen Zhang, Zhen Yang, Ke Dai, Bangdi Hu, Shiyu Xu, Yu Wang, Li Lei, Senyan Du, Qin Zhao, Xiaobo Huang, Rui Wu, Qigui Yan, Yiping Wang, Sanjie Cao, Yiping Wen

**Affiliations:** Research Center for Swine Diseases, College of Veterinary Medicine, Sichuan Agricultural University, Chengdu 611130, China; zhangyiwen@stu.sicau.edu.cn (Y.Z.);

**Keywords:** *Glaesserella parasuis*, Rab4b, *Gp*CDT, cytotoxicity

## Abstract

*Glaesserella parasuis* cytolethal distending toxin (*Gp*CDT) can induce cell cycle arrest and apoptosis. Our laboratory’s previous work demonstrated that GTPase 4b (Rab4b) is a key host protein implicated in *Gp*CDT-induced cytotoxicity. This study investigated the probable involvement of Rab4b in the process. Our study used CRISPR/Cas9 technology to create a Rab4b-knockout cell line. The results showed greater resistance to *Gp*CDT-induced cell cytotoxicity. In contrast, forced Rab4b overexpression increased *Gp*CDT-induced cytotoxicity. Further immunoprecipitation study reveals that *Gp*CDT may bind with Rab4b. In PK-15 cells, *Gp*CDT is transported to the early endosomes and late endosomes, while after knocking out Rab4b, *Gp*CDT cannot be transported to the early endosome via vesicles. Rab4b appears essential for *Gp*CDT-induced cytotoxicity in PK-15 cells.

## 1. Introduction

*Glaesserella parasuis,* a commensal pathogen, forms a symbiotic relationship with its host [1], which can cause Glässer’s disease. Clinical signs of the disease include fever, depression, anorexia, joint swelling with claudication, dyspnoea and central nervous system signs [2]. Recently, 15 serotypes of *Glaesserella parasuis* were recognized [3]. However, 20% of wild strains cannot be serotyped [4,5]. When the host is exposed to environmental stress or immunocompromised, it spreads to the lungs and causes pneumonia; it even enters the circulation and invades multiple tissues, causing the systemic character of Glässer’s disease, which is characterized by fibrinous polyserositis, arthritis, and meningitis [6].

Cytolethal distending toxin (CDT), produced by Gram-negative bacteria, induces significant cytotoxicity and inflammatory reactions in various cell types, and its presence has been demonstrated in more than 30 bacterial species [7,8]. CDT usually consists of proteins encoded by three consecutive reading frames located in the same manipulator to form an intact toxin in the form of a heterotrimer [9,10]. The main function of CDT is to damage the chromosomal DNA of eukaryotic cells, thus disrupting the normal cell division cycle, usually causing cell G2 phase arrest and lymphocyte apoptosis [11]. All three subunits of CDT possess a signal peptide, which is cleaved to form a mature protein subunit that self-assembles to form a complete trimeric toxin that is secreted into the external environment of the bacterium [12].

According to its structural characteristics, CDT is defined as an A-B2 exotoxin [7]. The CDT operon contains three genes; subunits CdtA and CdtC constitute the binding (B) unit and CdtB the active subunit [13]. CdtB has typical DNase characteristics, with five conserved amino acid residues consistent with the active site of mammalian type I deoxyribonuclease (DNase I) [14], which can damage the DNA of host cells [15]. The phosphatase activity of CdtB also causes the host cell to signal during the G2/M phase of the cell cycle, thereby blocking the cell cycle. CdtA and CdtC are responsible for binding to target cells and assisting Cdt B to enter eukaryotic cells [16]. Compared with other A-B toxins, CDT is unique in that it is transported to the Golgi apparatus and endoplasmic reticulum after the early and late endosomal stages. The transition from the endoplasmic reticulum to the nucleus does not occur through the traditional endoplasmic reticulum-associated degradation (ERAD) pathway, nor does the process of protein unfolding, and proteins are directly transferred to the nucleus to exert toxic effects [17,18].

Small GTPases of the Rab family are the main organizers of intracellular trafficking [19]. Rab proteins are localized to a variety of cellular organelles, including mitochondria, the endoplasmic reticulum, the Golgi apparatus, and the lysosome. Rab4b is an evolutionarily conserved GTP-binding protein that is mainly localized in lattice-encapsulated vesicles, early endosomes, and cycling endosomes and is an important regulator of endocytosis and cycling processes [20,21,22,23]. Existing research showed that mutations in the Rab4b protein can lead to cellular dysfunction and have been implicated in a variety of diseases [24,25]. Rab4b was identified as a host factor associated with *Gp*CDT infection in our previous study. This work is the first to examine Rab4b’s function and involvement in *GpCDT-induced* cells. This study will enrich the understanding of the mechanism by Rab4b to promote *GpCDT-induced* cytotoxicity in PK-15 cells.

## 2. Results

### 2.1. Construction of the Rab4b Knockout and Overexpressing Cell Lines

**Figure 1:**
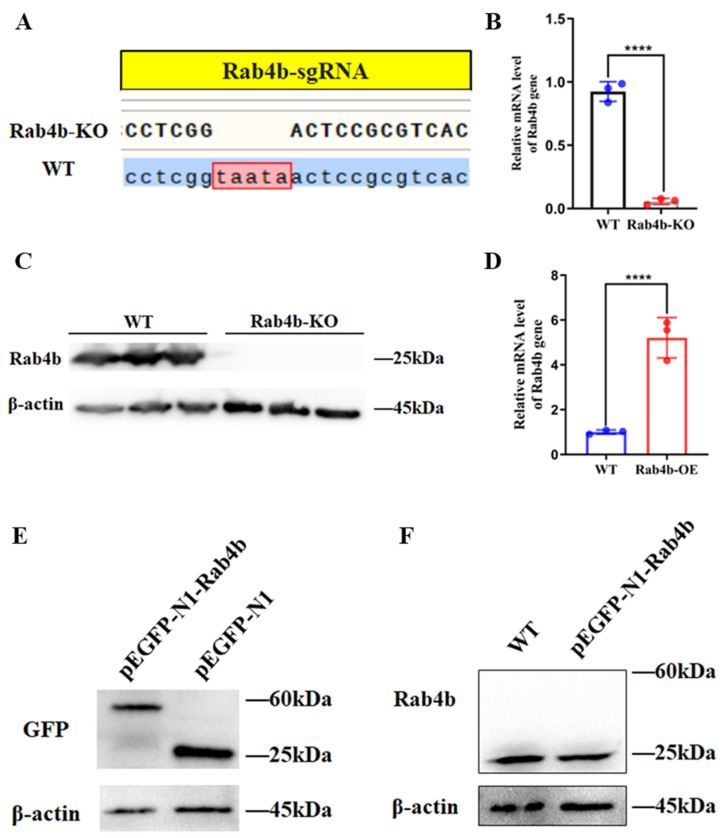
(**A**) Sequencing analysis of *Rab4b* gene in PK-15-Rab4b-KO cells. (**B**) Detection of *Rab4b* gene mRNA level by qRT-PCR. (**C**) Western blot detection of Rab4b protein expression level. (**D**) Overexpression of Rab4b in PK-15 cells detected by qRT-PCR. (**E**) Overexpression of Rab4b in PK-15 cells detected by Western blot using an anti-GFP antibody. (**F**) Overexpression of Rab4b in the PK-15 cells verified by Western blot using Rab4b antiserum (**** means *p* < 0.001).

As shown in Figure 1A, the CRISPR/Cas9-mediated knockout of the *Rab4b* gene was successful, as evidenced by the creation of a Rab4b-KO cell line with five bases missing from the original sequence in comparison to PK-15 cells. The Rab4b knockout monoclonal cell lines were screened by limited dilution, and Rab4b-KO cells were identified by qRT-PCR and Western blot. Genomic DNA extracted from monoclonal lines was subjected to PCR amplification and Sanger sequencing. This revealed five nucleotide deletions in the Rab4b-KO clone compared to wild-type parental cells (Figure 1A). The qRT-PCR results showed that the Rab4b gene mRNA level was almost undetectable on Rab4b-KO cells (Figure 1B). The Western blot analysis of Rab4b protein revealed similar results (Figure 1C), indicating that the screening of Rab4b knockdown cell lines was successful.

When the PK-15 cells in a 60 mm cell culture plate reached 50%, the pEGFP-N1-Rab4b and pEGFP-N1 were transfected into cells and the expression of Rab4b was detected by qRT-PCR and Western blot. The qRT-PCR assay results confirmed that Rab4b mRNA levels increased more than 3-fold in the Rab4b-OE cells (Figure 1D). The Western blot results showed that the expression of Rab4b was detected in PK-15 cells using anti-GFP as the primary antibody (60 kDa) (Figure 1E). However, no band at 60 kDa was observed when using a Rab4b antiserum as the primary antibody, indicating that the exogenous expression of Rab4b was not detected in PK-15 cells (Figure 1F).

### 2.2. Rab4b Knockout Inhibits GpCDT-Induced Cytotoxicity in PK-15 Cells

**Figure 2:**
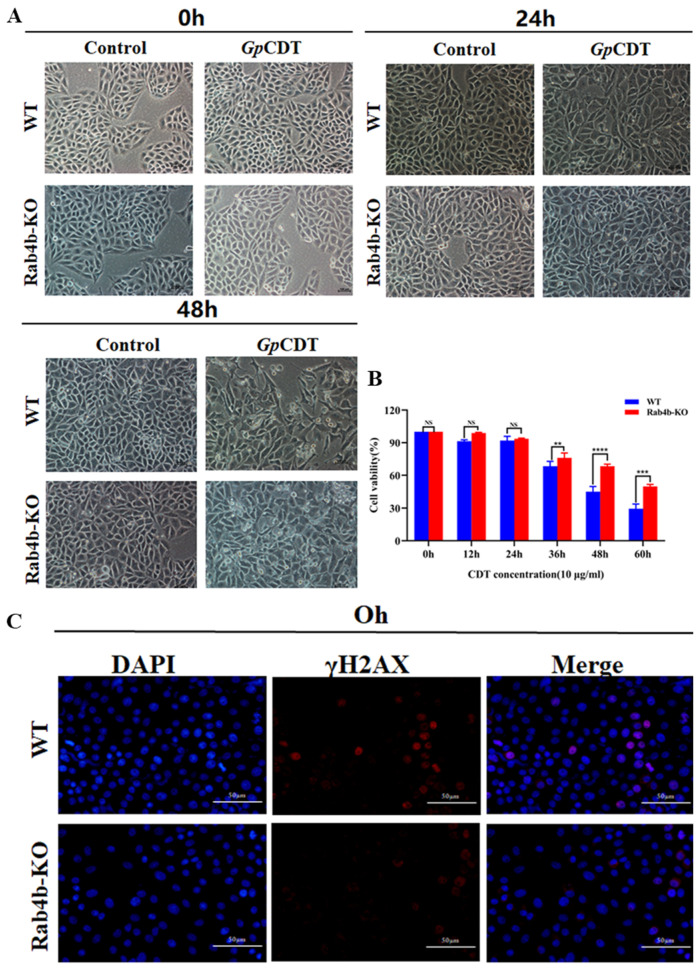

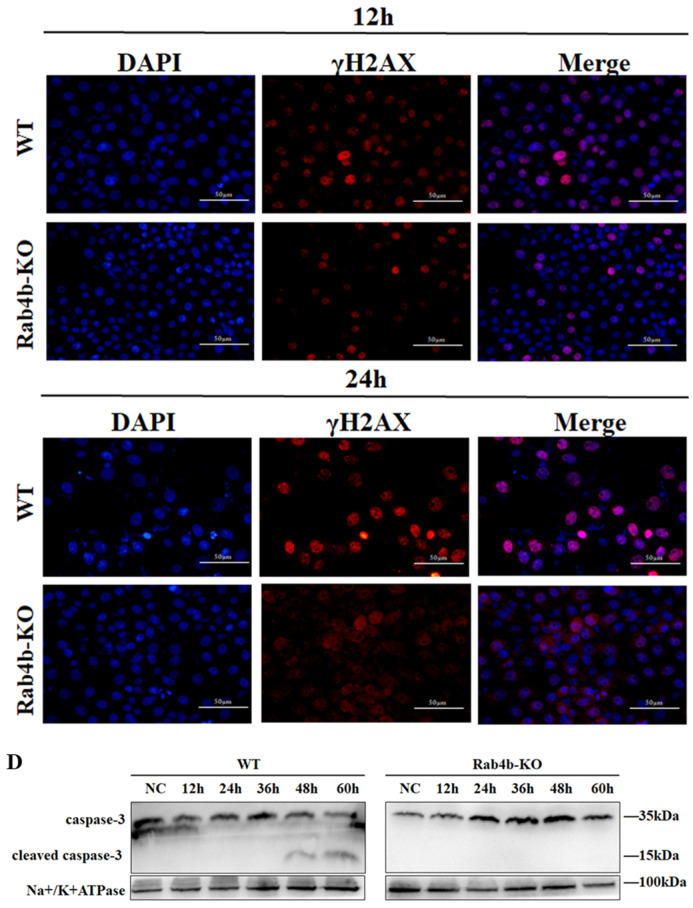

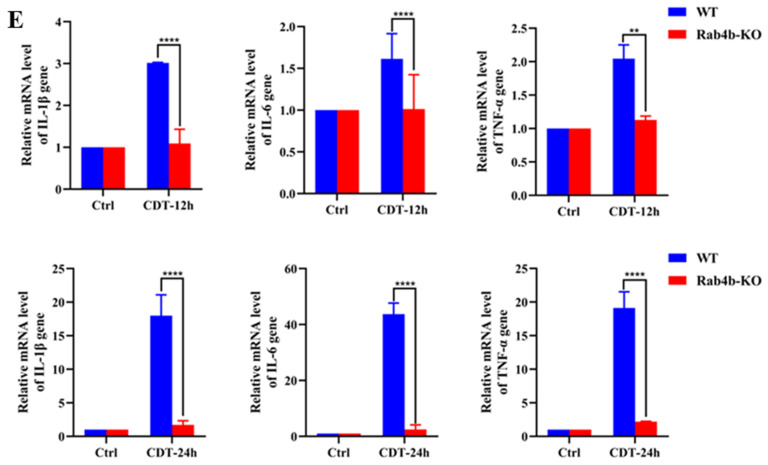
Knockout of Rab4b decreases *Gp*CDT-induced cytotoxicity in PK-15 cells. (**A**) Typical morphology of PK-15 cells and PK-15-Rab4b-KO cells treated with 10 μg/mL *Gp*CDT (400×). (**B**) Cell viability after exposure to 10 μg/mL *Gp*CDT for varying durations (0, 12, 24, 36, 48, and 60 h). (**C**) Utilizing immunofluorescence microscopy, the DNA damage signature γH2AX was identified after treating cells with *GpCDT* for 0, 12, and 24 h (** means *p* < 0.01, *** means *p* < 0.001, **** means *p* < 0.001, and ns means *p* > 0.05). (**D**) Caspase-3 levels were detected in PK-15 and PK-15-Rab4b-KO cells treated with *Gp*CDT. (**E**) Using qRT-PCR, the mRNA levels of IL-1β, IL-6, and TNF-α were measured.

To investigate the effect of Rab4b knockdown on PK-15 cells, we used *Gp*CDT to infect Rab4b-KO cells. Firstly, we observed that the morphology of PK-15 cells was dramatically changed when *Gp*CDT was treated. The gap widened, the number and density decreased, and the adherence to the wall was weakened. The intercellular junctions gradually vanished and became rounded, vacuolated, and detached, and there were also many dead cells floating. The knockout of Rab4b significantly attenuated the morphological changes (Figure 2A). As shown in Figure 2B, *Gp*CDT treatment induced significant cytotoxicity, as evidenced by CCK-8 optical density (OD) increase, while Rab4b knockout inhibited cell death (Figure 2B). Furthermore, PK-15 cells treated with *Gp*CDT had more γH2AX foci—a measure of DNA damage signal—than Rab4b-KO cells (Figure 2C). Our further studies in PK-15 cells showed a significant activation of *Gp*CDT-induced apoptosis, as evidenced by the activation of caspase-3. However, *Gp*CDT-induced apoptotic activation was significantly reduced in Rab4b-KO cells (Figure 2D). *Gp*CDT exposure causes cells to generate proinflammatory cytokines such as IL-1, IL-6, and TNF-α. To determine the targeted mRNA levels, we used qRT-PCR, which allowed us to explore the possible significance of Rab4b in the process. In PK-15 cells, there was an upregulation of IL-1, IL-6, and TNF-α mRNA levels. Of note, the knockout of Rab4b inhibited *Gp*CDT-induced mRNA expression (Figure 2E). Collectively, these findings indicate that the knockout of Rab4b markedly reduces the cytotoxic effects induced by *Gp*CDT in PK-15 cells.

### 2.3. Rab4b Overexpression Enhances GpCDT-Induced Cytotoxicity in PK-15 Cells

**Figure 3:**
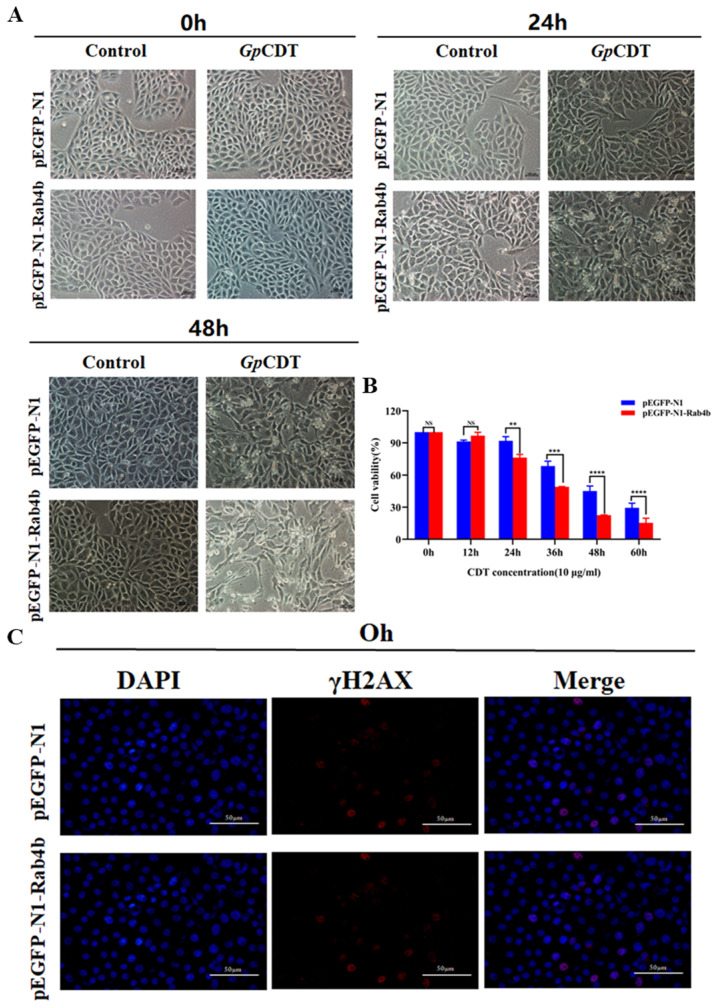

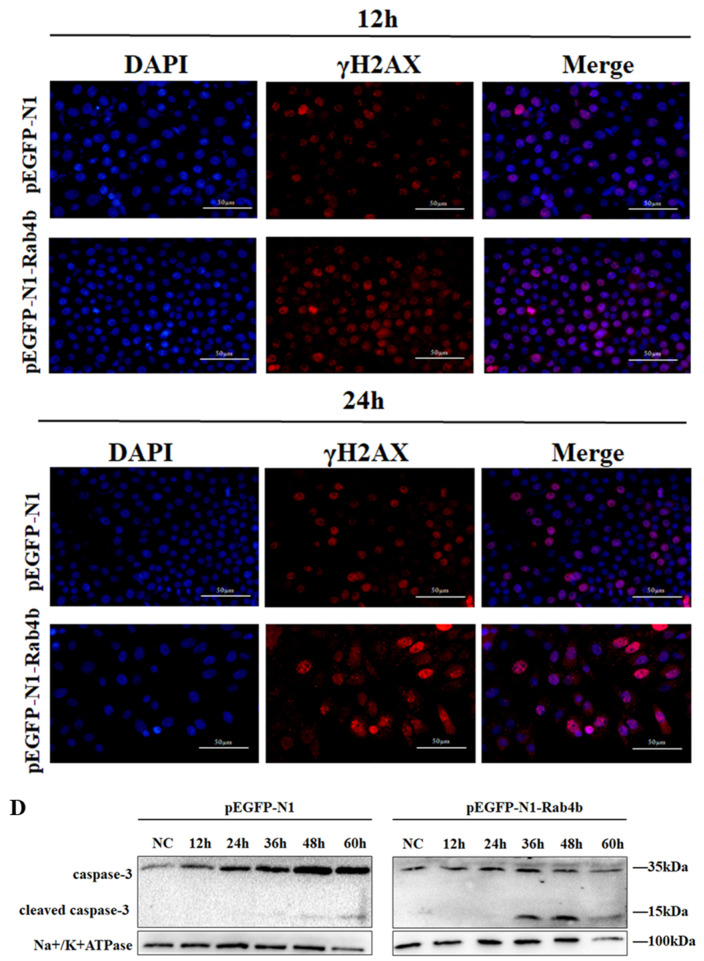

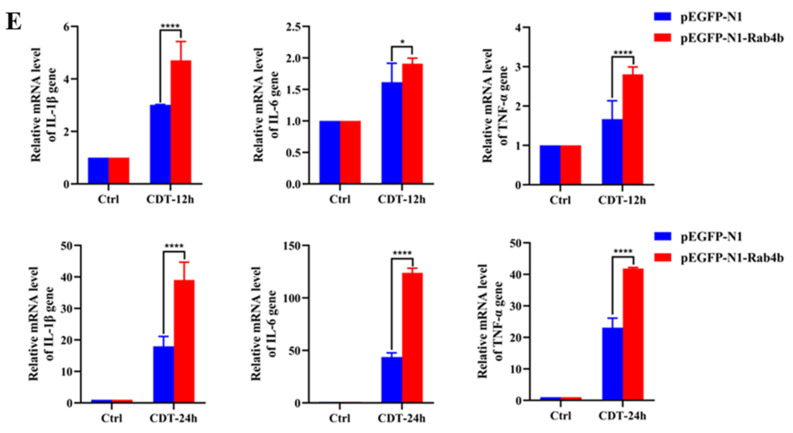
Rab4b overexpression enhances *Gp*CDT-induced cytotoxicity in PK-15 cells. (**A**) Typical morphology of PK-15 cells and PK-15-Rab4b-KO cells treated with 10 μg/mL *Gp*CDT for 0 h, 24 h, and 48 h (400×). (**B**) Cell viability after exposure to 10 μg/mL *Gp*CDT for varying durations (0, 12, 24, 36, 48, and 60 h). (**C**) Utilizing immunofluorescence microscopy, the DNA damage signature γH2AX was identified after treating cells with *Gp*CDT for 0, 12, and 24 h (* means *p* < 0.05, ** means *p* < 0.01, *** means *p* < 0.001, **** means *p* < 0.001, and ns means *p* > 0.05). (**D**) Caspase-3 levels were detected in PK-15 and PK-15-Rab4b-KO cells treated with *Gp*CDT. (**E**) Using qRT-PCR, the mRNA levels of IL-1β, IL-6, and TNF-α were measured.

The above results show that Rab4b knockout (Figure 2) potently inhibited *Gp*CDT-induced cytotoxicity in PK-15 cells. The overexpression of Rab4b resulted in significantly increased cell distension (Figure 3A). Crucially, Rab4b-OE cells responded to *Gp*CDT by exhibiting considerably higher levels of *Gp*CDT-induced cell death (Figure 3B), DNA damage (Figure 3C), and apoptosis (Figure 3D) as compared to PK-15 cells. Moreover, the findings of qRT-PCR demonstrated that *Gp*CDT significantly increased the mRNA expression of IL-1β, IL-6, and TNF-α in Rab4b-OE cells (Figure 3E). These findings imply that Rab4b overexpression increased the cytotoxic effects of *Gp*CDT. In summary, our results demonstrate the critical role Rab4b plays in the cytotoxicity that *Gp*CDT induces in PK-15 cells.

### 2.4. Further Validation of Interactions between Rab4b and GpCDT Proteins

**Figure 4:**
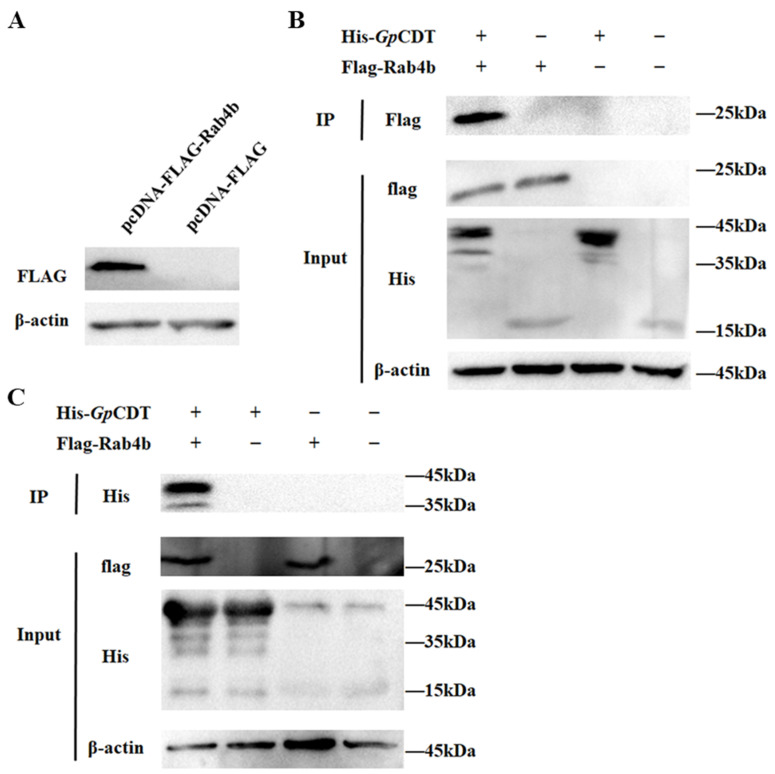
Interaction of Rab4b and *Gp*CTD protein was detected by coimmunoprecipitation. (**A**) Expression of the *Rab4b* gene on HEK293T cells detected by Western blot. (**B**) Immunoblot of His-*Gp*CDT protein and host factor Rab4b precipitated using anti-Flag Mab. (**C**) Immunoblot of host factor Rab4b and His-*Gp*CDT protein precipitated using anti-His MAb. +: add the target protein; −: add the tag protein.

Coimmunoprecipitation assays were used to see if *Gp*CDT proteins interact with Rab4b. The pcDNA-3.1-Flag-Rab4b and pcDNA-3.1-Flag transfection were performed on HEK293T cells. Western blotting was used to confirm that Rab4b was expressed (Figure 4A). Coimmunoprecipitations were performed using anti-Flag and anti-His antibodies to capture protein complexes. The Western blot results showed that Flag-tagged Rab4b and His-tagged *Gp*CDT were detected in the immunoprecipitated complexes, suggesting an interaction between Rab4b and *Gp*CDT proteins (Figure 4B). Therefore, we speculated that Rab4b is crucial to the infection of PK-15 cells by *Gp*CDT.

### 2.5. Rab4b Affects the Vesicle Transport of GpCDT in PK-15 Cells

**Figure 5:**
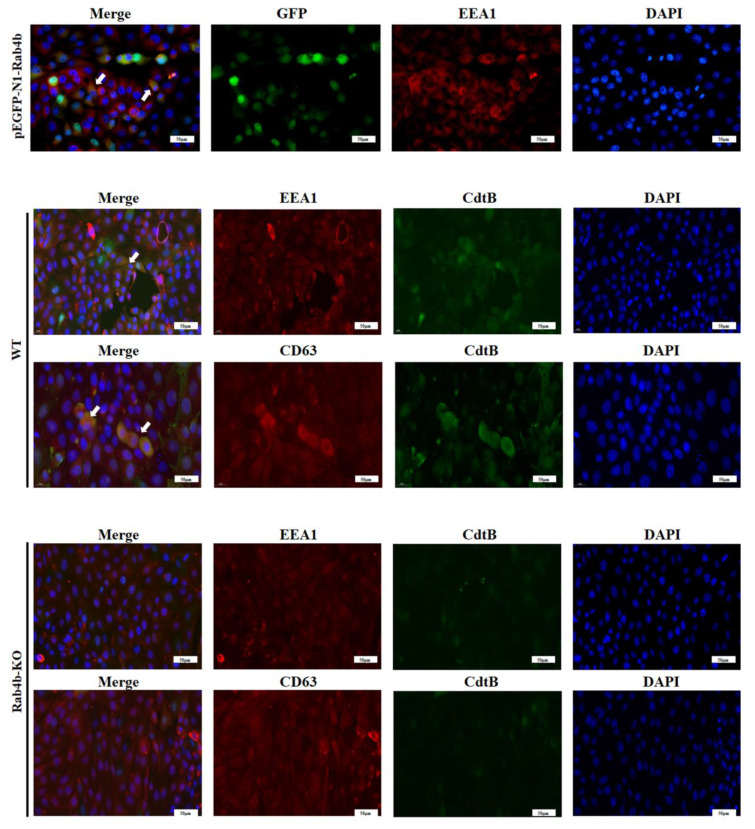
(**A**) Images of GFP-Rab4b (green) localized to EEA1 (red). (**B**,**C**) The IFA results for the PK-15 and PK-15-Rab4b-KO cells treated with *Gp*CDT. Red fluorescence indicates CdtB and green fluorescence indicates the organelle marker. EEA1: marker of early endosome; CD63: marker of late endosome; blue fluorescence indicates nuclear; arrow: where the two fluoresces are superimposed (400×, scale bar = 50 μm).

Localized to early endosomes, rab4b controls the production of recycling vesicles [26]. As shown in Figure 5A, endogenous EEA1-labeled early endosomes co-localized with GFP-Rab4b in PK-15 cells (Figure 5A). It has been demonstrated in several cell types that CdtB traffics to early and late endosomes after internalization. Following treatment with *Gp*CDT, PK-15 cells show CdtB fluorescence co-localized with both early endosomes (EEA1) and late endosomes (CD63) (Figure 5B). Nonetheless, in Rab4b-KO cells, we were unable to detect any CdtB fluorescence co-localization with early endosomes (EEA1) and late endosomes (CD63) (Figure 5C). These findings provide more evidence that Rab4b can impact *Gp*CDT vesicle trafficking. All of the experimental data support our theory that Rab4b influences the vesicle transport of *Gp*CDT in PK-15 cells, hence contributing to the cytotoxicity that *Gp*CDT induces.

## 3. Discussion

*Glaesserella parasuis* is the causative agent of swine Graves’ disease, which is highly morbid and often secondary to virulent infections in pigs. It is a conditionally pathogenic organism that colonizes the upper respiratory tract of healthy pigs and causes a systemic inflammatory response [1]. CDT, produced by Gram-negative bacteria, induces significant cytotoxicity and inflammatory reactions in various cell types, such as T-cell and B-cell lines [27,28]. To date, only a few studies have reported the effects of Rab4b on the cytotoxic effects of bacterial toxin proteins. The current study’s findings imply that Rab4b may be fundamental in mediating the effects of *Gp*CDT. Some scholars have shown that Rab4b transcription is upregulated 12-fold after 4 h of *Corynebacterium* infection [29]. In this study, the knockout of Rab4b weakened the cytotoxicity, DNA damage, cell apoptosis, and inflammatory response of *Gp*CDT, whereas the overexpression of Rab4b enhanced these biological effects of *Gp*CDT. However, the specific mechanism by which Rab4b mediates *Gp*CDT cytotoxicity needs to be further explored.

It is well known that CdtB, the active subunit of CDT, blocks the phosphatidylinositol 3-kinase (PI3K)-AKT signaling pathway, which results in apoptosis and cell death [28,30,31]. Previous research has suggested that CdtB may also induce double-strand breaks (DSBs), which can lead to cell death. Significant inflammatory reactions may also be triggered by CDT [32], and additional pathways for mediating CDT-induced cytotoxicity have also been suggested [31]. We proved the existence of a potential direct interaction between Rab4b and *Gp*CDT. More research is therefore required to verify the connection between Rab4b and these suggested pathways by *Gp*CDT.

Perrin et al. found that as a vital host factor in cells, Rab4b co-localized with early endosomes in HeLa cells and regulated the recovery and aggregation of TfR through its influence on vesicle transport [22]. And Kaddai et al. demonstrated that Rab4b colocalizes with early endosomes in the adipocytes of obese diabetic humans and mice, and is involved in the vesicular transport of glucose transporter GLUT4 [33]. Hence, we focused on the relationship between Rab4b and the vesicle transport process of *Gp*CDT in PK-15 cells. The current study’s findings indicate that Rab4b co-localized with early endosomes in PK-15 cells, indicating that it is most likely involved in the vesicle transport of *Gp*CDT.

*Gp*CDT is known to need to be transported to the nucleus to perform its DNA-damaging effects, causing cell cycle arrest and cell death [11]. Rab4b is located mainly in early endosomes and circulating endosomes. As an important regulatory factor in the intracellular vesicle transport process, Rab4b can affect the intracellular vesicle transport process of extracellular substances ingested by cells [34]. Rab4b exerts its endosomal sorting function to mediate the vesicle transport of *Gp*CDT so that the CdtB subunit of *Gp*CDT can be accurately transported to the nucleus to perform its biological function. Indirect immunofluorescence can determine the vesicle transport process of CDT after uptake by cells. Existing studies have proposed that CdtB fluorescence was co-localized with early endosomes and Golgi apparatus, but it did not show any co-localization of CdtB fluorescence with human macrophage mitochondria and lysosomes [11]. In this work, we employed indirect immunofluorescence to observe the colocalization of *Gp*CDT with early and late endosomes following cellular uptake. However, in Rab4b -KO cells, *Gp*CDT did not colocalize with early endosomes, indicating that the knockout of Rab4b resulted in the inhibition of *Gp*CDT vesicle transport. *Gp*CDT is not transported to early endosomes through vesicles and thus cannot reach the nucleus to perform its biological function, resulting in weakened *Gp*CDT-induced cytotoxicity. The current study’s findings imply that Rab4b may be key in modulating GpCDT-induced behaviors.

In conclusion, our previous laboratory work identified Rab4b as a host factor involved in *Gp*CDT-induced cytotoxicity. In this study, we used WT, Rab4b-KO, and Rab4b-OE cells, along with CCK-8, indirect immunofluorescence, qRT-PCR, and Western blot assays, to investigate Rab4b’s role in *Gp*CDT-induced cytotoxicity. The knockout of Rab4b made cells more resistant to *Gp*CDT-induced cell cytotoxicity, while forced Rab4b overexpression increased *Gp*CDT-induced cytotoxicity. Furthermore, we found that the knockout of Rab4b resulted in *Gp*CDT not being able to reach the late endosomes. We suspect that Rab4b promotes *Gp*CDT-induced cytotoxicity by controlling the vesicle transport of *Gp*CDT in PK-15 cells. We also demonstrated that Rab4b may interact with *Gp*CDT. We will further investigate the relationship between *Gp*CDT subunits and Rab4b, as well as the key action sites. This study establishes a foundational basis for the in-depth investigation of the specific mechanisms through which Rab4b modulates the progression of *Gp*CDT-induced cytotoxicity. These results provide better methods for treating *Glaesserella parasuis* infections and advance our knowledge of the pathogenic mechanism of both *Gp*CDT and *Glaesserella parasuis*.

## 4. Conclusions

For the first time, our study confirmed that Rab4b plays a crucial role in *Gp*CDT-induced cytotoxicity. The knockout of Rab4b makes cells more resistant to *Gp*CDT-induced cell cytotoxicity, while forced Rab4b overexpression increased *Gp*CDT-induced cytotoxicity. Furthermore, Rab4b has a potential direct interaction with *Gp*CDT and co-localized with early endosomes in PK-15 cells. These findings may prove that Rab4b appears crucial for *Gp*CDT-induced cytotoxicity in PK-15 cells because it regulates the vesicle transit of *Gp*CDT. This work establishes a framework for further investigation into the precise mechanism by which Rab4b affects the progression of *Gp*CDT infection.

## 5. Materials and Methods

### 5.1. Cells, Plasmids, and Growth Conditions

Pig kidney (PK-15) cells and human embryo kidney (HEK-293T) cells were grown in DMEM (Gibco, Carlsbad, CA, USA) supplemented with 10% fetal bovine serum (Gibco, Carlsbad, CA, USA). The cells were subcultured upon reaching 90% confluence and incubated in a 37 °C incubator containing 5% CO_2_.

Table 1 lists the plasmids and bacterial strains that were utilized in this investigation. All strains grown in broth were cultured with shaking at 220 r/min at 37 °C.

### 5.2. Expression of GpCDT and Mouse Antiserum

The pET-cdtA, pET-cdtB, and pET-cdtC plasmids constructed and preserved in the laboratory were resuscitated and expanded. CdtA, CdtB, and CdtC were subsequently expressed according to the established induction conditions. Then, CdtA, CdtB, and CdtC were mixed and allowed to stand at 4 °C overnight for subsequent studies [35]. The induction conditions for the His-tag protein were induced with 1.2 mM IPTG for 16 h at 18 °C [36].

Rab4b antiserum was produced by immunizing mice with the C terminal peptide of Rab4b coupled with KLH [22]. CdtB antiserum was produced by immunizing subcutaneously with 0.1 mg of rCdtB and supplemented with 20 µL of the water adjuvant Montanide Gel 01 (SEPPIC, Paris, France). Blood was then collected overnight at 4 °C and serum was collected [35].

### 5.3. Knockout of Rab4b Gene and Overexpression of Rab4b Protein

The Rab4b gene knockout in PK15 cell lines (PK15-Rab4b-KO) was performed by using CRISPR/Cas-9 gene-editing technology. In brief, a pair of sgRNA targeting the swine Rab4b gene was designed by using the CRISPR online editing website (http://chopchop.cbu.uib.no/), and then the sgRNA was inserted into the lentiCRISPR-V2 plasmid to target Rab4b (Table 2). The recombinant plasmid, psPAX2: pMD2.G = 5:3:2, was co-transfected into HEK293T cells using Lipofectamine 3000 (Invitrogen, Carlsbad, CA, USA). The supernatant was collected 48 h later. When the PK-15 cells in T25 cell culture flasks grew to 50%, they were infected with lentivirus, and 8 μg/mL polybrene was added at the same time for 3–6 days.

The eukaryotic expression vector pEGFP-N1-Rab4b was created by cloning the Rab4b gene from PK-15 cells and inserting it into the enzymatically cut pEGFP-N1 vector (Table 2). When PK-15 cells in 60 mm cell culture dishes reached 50% confluence, 4 μg of the pEGFP-N1-Rab4b plasmid was transfected into the cells with Lipofectamine^®^ 3000 Transfection Kit (Invitrogen, Carlsbad, CA, USA). Thirty-six hours later, the cells were harvested, and the successful overexpression of Rab4b was extracted by Western blotting. At the same time, the same amount of empty plasmid pEGFP-N1 was transfected as a control.

### 5.4. Microscopy Imaging

PK-15 cells were inoculated in 6-well plates at a 5 × 10^5^ cells/well density. After the applied treatments, cytopathic changes in PK-15 cells were observed and captured by light microscopy (Olympus America, Center Valley, PA, USA) every 12 h for 60 h after infection.

### 5.5. Cell Viability Assay

Cytotoxicity induced by *Gp*CDT was assessed using CCK-8 (Beyotime Biotechnology, Shanghai, China). The cells were treated with *Gp*CDT (10 µg/mL). After washing with PBS, the cells were incubated for 1 h at 37 °C with 10 μL CCK-8 reagent per well. Absorbance was measured at 450 nm using an enzyme marker (Bio-Rad, Hercules, CA, USA).

### 5.6. Western Blotting

Cells were treated as described. Both cells and supernatants were harvested via a cell scraper at 0, 12, 24,36, 48, and 60 h. Samples were separated on 12.5% SDS-PAGE and then transferred to PVDF membranes. The membranes were blocked with 5% nonfat dry milk and then incubated with primary antibody (anti-caspase-3 rabbit mAb or anti-sodium potassium ATPase rabbit mAb). The membranes were then washed and incubated with the secondary antibody. As a final step, images were captured using ChemiDoc TM MP Imaging System (BioRad, Hercules, CA, USA).

### 5.7. Indirect Immunofluorescence

To examine γH2AX foci, the cell samples were washed three times with PBS, fixed, permeabilized, and blocked in 3% nonfat dry milk. The cells were then incubated with anti-Phospho-Histone H2AX rabbit mAb at 4 °C overnight, followed by incubation with FITC-conjugated secondary antibodies for 1 h and DAPI for 15 min at room temperature. Cells were observed under a fluorescence microscope (Olympus BX63, Tokyo, Japan).

The study on trafficking was carried out as follows. PK-15 and PK-15-Rab4b-KO cells were exposed to 10 µg/mL *Gp*CDT holotoxin at 4 °C for 30 min to facilitate binding to the cytomembrane, followed by incubation at 37 °C for 45 min to stimulate uptake. The next steps are described above.

### 5.8. Quantitative Real-Time PCR

Cell samples were taken from 24-well cell culture plates after the recommended treatment, and total RNA was extracted from the cells using a Total RNA Isolation Kit (Sangon Biotech, Shanghai, China). Then, we used the HiScript III RT SuperMix (Vazyme, Nanjing, China) for reverse transcription. To determine the RNA expression level, a 20 µL mixture containing diluted cDNA was analyzed for qRT-PCR using ChamQ SYBR Color qPCR Master Mix (Vazyme, Nanjing, China) in a Bio-rad CFX96 System (Bio-rad, Hercules, CA, USA) [35]. Table 2 lists the primer sequences that were employed.

### 5.9. Creation of Plasmids and Coimmunoprecipitation Assay

The plasmid for the expression of pCDNA3.1-flag-Rab4b was made and kept in our lab. The pCDNA3.1-flag-Rab4b plasmid was transfected into HEK293T cells in order to examine an association between Rab4b and *Gp*CDT. Cell supernatant was collected 24 h later. After being cleaned with cell lysate, protein A/G beads were added to the supernatant or *Gp*CDT and gently rocked for four hours at 4 °C. Subsequently, *Gp*CDT or supernatant was added and the mixture was gently rocked for four hours at 4 °C in the incubator. Western blotting was used to test the beads after they had been cleaned four times with cold cell lysate.

### 5.10. Statistical Analysis

The experiments were carried out three times in duplicate. The software used for statistical analysis was GraphPad Prism version 8.0. Statistical comparisons between the two groups were performed using unpaired *t*-tests. *p* < 0.05 was considered to indicate a statistically significant difference. * *p* < 0.05 was considered statistically significant; ** *p* < 0.01, *** *p* < 0.001, and **** *p* < 0.001 indicated extremely significant differences.

## Figures and Tables

**Table 1:** Bacterial strains and plasmids used in this study.

Strain or Plasmid	Relevant Characteristics	Source
*E. coli* DH5α	F^−^ endA1 glnV44 thi-1 recA1 relA1 gyrA96 deoR nupG purB20 φ80dlacZΔM15 Δ(lacZYA-argF) U169, hsdR17(rK^−^mK^+^), λ^−^	Biomed
pET-cdtA	A 624 bp cdtA CDS in pET-32a (+)	Laboratory collection
pET-cdtB	A 768 bp cdtB CDS in pET-32a (+)	Laboratory collection
pET-cdtC	A 471 bp cdtC CDS in pET-32a (+)	Laboratory collection
pET-32a (+)	Prokaryotic expression vector	Laboratory collection
pMD2.G	Lentivirus envelope plasmid	Laboratory collection
pSPAX2	Lentivirus envelope plasmid	Laboratory collection
pLentiCRISPR V2	sgRNA carrier plasmid	Laboratory collection
pEGFP-N1	Overexpression plasmid	Laboratory collection
pcDNA3.1-Flag	Eukaryotic expression vector	Laboratory collection
pcDNA3.1-Flag-Rab4b	Overexpression plasmid	Laboratory collection

**Table 2:** Primers used in this study.

Gene	Primer Direction	Sequence (5′–3′)	Size (bp)
Rab4b-sgRNA	Forward	CACCGTGACGCGGAGTTATTACCG	24 bp
	Reverse	AAACCGGTAATAACTCCGCGTCAC	
Rab4b-KO	Forward	CACAATCGGCGTGGAGTT	176 bp
	Reverse	AGTTGTAAGTCTCCCGGCTGT	
Rab4b-KO	Forward	CACAATCGGCGTGGAGTT	176 bp
	Reverse	AGTTGTAAGTCTCCCGGCTGT	
pEGFP-N1-*Rab4b*	Forward	GACTCAGATCTCGAGGCCACCATGGCTGAGACCTACGACTTCC	682 bp
	Reverse	GTACCGTCGACTGCAGAATTCCGCAGCCACAGGGCTGAGG	
IL-6-sus	Forward	GGGACTGATGCTGGTGACAA	147 bp
	Reverse	TCCACGATTTCCCAGAGAACA	
TNF-α-sus	Forward	CGTCAGCCGATTTGCTATCT	184 bp
	Reverse	CTTGGGCAGATTGACCTCAG	
β-actin-sus	Forward	CTTCCTGGGCATGGAGTCC	201 bp
	Reverse	GGCGCGATGATCTTGATCTTC	

## Data Availability

The data presented in this study are available upon request from the corresponding author.
